# Childhood adversities and bipolar disorder: a neuroimaging focus

**DOI:** 10.1017/S2045796021000834

**Published:** 2022-02-03

**Authors:** Niccolò Zovetti, Cinzia Perlini, Paolo Brambilla, Marcella Bellani

**Affiliations:** 1 Department of Neurosciences, Biomedicine and Movement Sciences, Section of Psychiatry, University of Verona, Verona, Italy; 2 Department of Neurosciences, Biomedicine and Movement Sciences, Section of Clinical Psychology, University of Verona, Verona, Italy; 3 Department of Neuroscience and Mental Health, Fondazione IRCCS Ca’ Granda Ospedale Maggiore Policlinico, Milan, Italy; 4 Department of Pathophysiology and Transplantation, University of Milan, Milan, Italy

**Keywords:** bipolar disorder, brain imaging, childhood adversity, childhood trauma, early-life events

## Abstract

Early-life adverse events or childhood adversities (CAs) are stressors and harmful experiences severely impacting on a child's wellbeing and development. Examples of CAs include parental neglect, emotional and physical abuse and bullying. Even though the prevalence of CAs and their psychological effects in both healthy and psychiatric populations is established, only a paucity of studies have investigated the neurobiological firms associated with CAs in bipolar disorder (BD). In particular, the exact neural mechanisms and trajectories of biopsychosocial models integrating both environmental and genetic effects are still debated. Considering the potential impact of CAs on BD, including its clinical manifestations, we reviewed existing literature discussing the association between CAs and brain alterations in BD patients. Results showed that CAs are associated with volume alterations of several grey matter regions including the hippocampus, thalamus, amygdala and frontal cortex. A handful of studies suggest the presence of alterations in the corpus callosum and the pre-fronto-limbic connectivity at rest. Alterations in these regions of the brain of patients with BD are possibly due to the effect of stress produced by CAs, being hippocampus part of the hypothalamus–pituitary–adrenal axis and thalamus together with amygdala filtering sensory information and regulating emotional responses. However, results are mixed possibly due to the heterogeneity of methods and study design. Future neuroimaging studies disentangling between different types of CAs or differentiating between BD sub-types are needed in order to understand the link between CAs and BD.

Early life adverse events or childhood adversities (CAs) refer to a wide spectrum of stressors and detrimental experiences including physical, sexual and psychological abuses and parental neglect deeply impacting on a child's wellbeing with potential unfavourable effects on development (Browne and Winkelman, 2007). Examples of early life adverse events include parental loss, maltreatment and bullying, all harming in some form the child's physical and emotional wellbeing and health (Butchart, 2006). According to recent studies and estimates, approximately 10% of children every year are victims of some form of maltreatment, with neglect, physical and sexual abuses being the most common (Font and Maguire-Jack, 2020).

CAs are known to be an important risk factor for the development of several psychiatric conditions including schizophrenia and bipolar disorder (BD); recent studies suggested that CAs are significantly associated with the onset of more than 40% of all childhood psychiatric disorders and with more than 25% of adult psychiatric disorders (Green *et al*., 2010). For example, in a recent study by Tomassi *et al*. (2017) the authors found that sexual abuse was significantly associated with a diagnosis of affective psychosis and substance abuse (Tomassi *et al*., 2017). Other studies indicated that CAs are consistently associated with BD and that the severity of its clinical manifestations is correlated with the number and severity of CAs (Etain *et al*., 2013; Aas *et al*., 2016). Specifically, some studies support the idea that CAs represent a risk factor for the emergence of BD, showing that the prevalence of CAs is higher in BD patients *v*. healthy controls (HC) for all types of CAs except for parental loss (Marangoni *et al*., 2016; Palmier-Claus *et al*., 2016; Perlini *et al*., 2020). For example, in a large recent study by our group (Pedrini *et al*., 2021) we showed that, in BD patients, CAs are associated with worse clinical manifestations during euthymic phases and that a considerable number of patients report multiple CAs during their lifetime with neglect being more frequent *v*. HC (Pedrini *et al*., 2021). Moreover, the number and severity of CAs has been shown to influence clinical factors such as age of onset, risk for suicide, frequency of mood episodes and response to treatment (Aas *et al*., 2016; Nemeroff, 2016).

Several biopsychosocial models have been proposed for the integration of genetic vulnerabilities and socio-environmental factors in psychotic and bipolar disorders including the multiple-hit models of susceptibility (Aas *et al*., 2016). Briefly, these models take into account the wide heterogeneity of individuals suffering from CAs and integrate genetic susceptibilities, environmental factors and their complex interactions aiming at understanding why only some of them develop psychiatric conditions, such as BD. According to these models, different neurodevelopmental trajectories are determined by the interplay between all these variables ultimately giving rise to BD only in a relatively small portion of individuals. For example, in a recent work by Jaworska-Andryszewska and Rybakowski, the authors suggested that CAs can affect specific regions of the central nervous system (e.g. hippocampus and amygdala) associated with the development of mood disorders (Jaworska-Andryszewska and Rybakowski, 2019). This is due to the interaction of environmental and genetic factors, the most important being the serotonin transporter gene and the FKBP5 gene. However, the exact mechanisms and trajectories of these models are still unclear including their neurobiological correlates.

Increasing evidence from neuroimaging studies suggest that CAs are associated with multiple brain alterations in healthy individuals mainly localised in the prefrontal cortex, hippocampus, amygdala, corpus callosum (CC) and other white matter (WM) structures (McCrory *et al*., 2011; Lim *et al*., 2014; Teicher *et al*., 2016). Similarly, early life traumas have been suggested to alter WM structures, grey matter (GM) volumes and functional connectivity of several brain regions in patients suffering from psychosis (Cancel *et al*., 2019). However, little is known about the possible effects of CAs in the BD brain. Considering the potential impact of CAs on brain development and their relevance in the development and characterisation of many clinical features of BD, it is crucial to better elucidate on the neurobiological correlates of CAs in BD. Therefore, this paper reviewed existing literature discussing the association between CAs and brain alterations in BD patients.

The data search was conducted on the PubMed, Scopus and Google Scholar databases. The following query was used for the search: (‘bipolar disorder’) AND (‘imaging’ OR ‘neuroimaging’ OR ‘magnetic resonance imaging’ OR ‘magnetic resonance’ OR ‘electroencephalography’ OR ‘Positron Emission Tomography’ OR ‘diffusion tensor imaging’ OR ‘brain’ OR ‘grey matter’ OR ‘gray matter’ OR ‘white matter’) AND (‘childhood abuse’ OR ‘childhood maltreatment’ OR ‘childhood trauma’ OR ‘early life adverse events’ OR ‘early life stress’ OR ‘adverse events’ OR ‘neglect’). Preclinical studies and case-report were excluded. A flow diagram illustrating the studies selection process is presented in Fig. 1. The literature search retrieved 136 records. After title and abstract screening, 121 articles were excluded because they clearly did not meet the inclusion criteria. The remaining 14 studies were included in this review after a full-text review. Sample characteristics and neuroimaging findings from each study are shown in Table 1. The data search was conducted on September 2021.

**Fig. 1.**
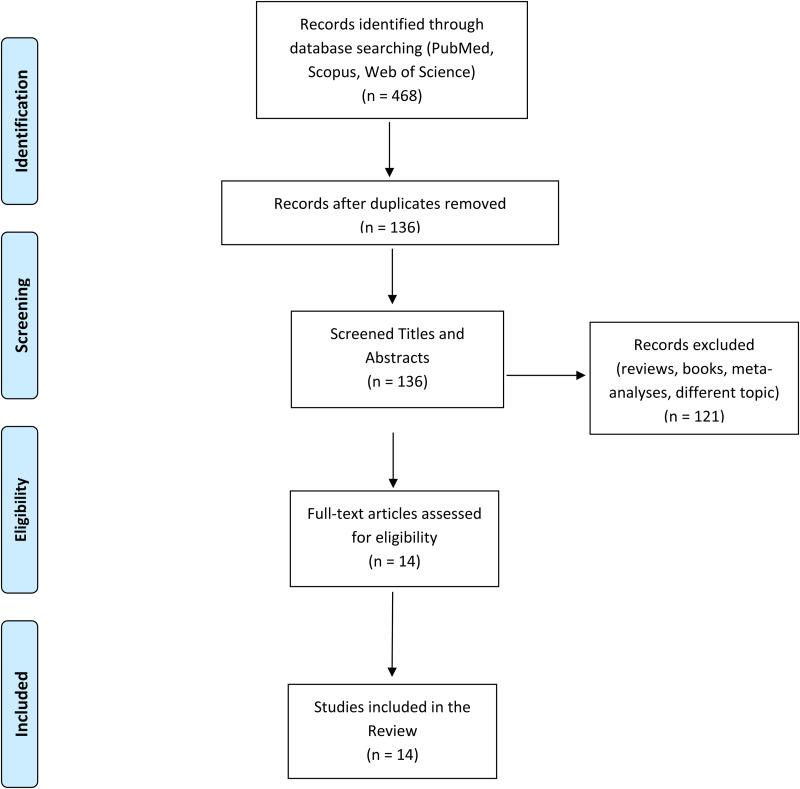
A graphic representation of our search query including the exact number of studies found and excluded.

**Table 1.** Studies investigating the neurobiological correlates of early adversities in bipolar disorder

Reference	Participants (% f)	Mean age (s.d.)	Adversity assessment tool	Neurobiological correlates measured	Results
Aas *et al*. (2013)	106 BD or SCZ (51%)	32.6 (10.8)	CTQ	GM volume	Patients were divided into two groups, Methionine or Valine allele carriers. Methionine carriers with a history of childhood sexual abused showed lower hippocampus volume and larger lateral ventricles
Aas *et al*. (2014)	108 BD or SCZ (52%)	31.9 (11.2)	CTQ short form	GM volume	Val66met patients with high levels of childhood trauma showed lower BDNF mRNA levels and reduced hippocampal and dentate gyrus volumes when compared with other patients
Bücker *et al*. (2014)	23 BD with trauma (56%), 30 BD without trauma (53%), 16 HC (50%)	22 (3.1), 23.5 (5.3), 21.5 (4.4)	CTQ	WM volume	Compared to patients without trauma, BD patients with a history of trauma were characterised by lower CC volumes. A significant correlation between CTQ scores and total CC volume in BD patients with trauma was found
Duarte *et al*. (2016)	39 BD-I (58%), 20 HC (55%)	40.3 (10.2), 37.4 (10.2)	CTQ	GM volume	Compared to HC, BD-I patients had significant negative correlations between childhood maltreatment levels and GM volumes in the right dorsolateral PFC, physical/emotional neglect and GM volume in the thalamus
Poletti *et al*. (2016)	96 SCZ (30%), 206 BD (65%), 136 HC (50%)	37.2 (9.3), 46.1 (12.9), 33.3 (12.9)	RFQ	GM volume	Patients and HC were divided into two groups, low and high adverse childhood experiences. When compared with HC, BD with high adverse childhood experiences showed lower GM volumes in the OFC and thalamus
Souza-Queiroz *et al*. (2016)	32 BD (53%), 47 HC (37%)	35.8 (11.2), 36.4 (11.3)	CTQ short form	GM volume, BOLD signal, WM fractional anisotropy	Correlation analyses between childhood trauma levels and several brain indices showed that BD patients, when compared to HC, were characterised by lower amygdala volume, lower prefronto-limbic connectivity at rest and lower fractional anisotropy in the uncinate fasciculus
Aas *et al*. (2017)	101 SCZ or BD (44%)	31.9 (10.1)	CTQ	BOLD signal	Patients with a history of childhood trauma showed stronger activations in the right angular, supramarginal, middle temporal gyri and lateral occipital cortex during an emotion recognition task
Janiri *et al*. (2017)	105 BD (49%), 113 HC (45%)	43.3 (11.4), 45.8 (12.2)	CTQ short form	GM volume	Patients and HCs were divided into two groups, with and without childhood traumas. Compared to HC, BD patients with a history of childhood trauma showed greater GM volumes in the amygdala and hippocampus
Stevelink *et al*. (2018)	251 BD (51%), 163 HC (50%)	48.1 (12.2), 45.1 (14.8)	CTQ short form	WM fractional anisotropy	In BD patients, higher levels of childhood abuse and trauma were negatively correlated with fractional anisotropy in the anterior, posterior, superior corona radiata and in the CC when compared with HCs
Aas *et al*. (2019)	254 BD (60%), 589 SCZ (40%), 603 HC (49%)	33.5 (11.7), 30 (9.8), 32.8 (9.6)	CTQ	BDNF levels	Compared to HC, patients were characterised by lower BDNF levels. Lower levels were found in patients reporting childhood sexual abuse
Janiri *et al*. (2019)	56 BD-I (90%), 49 BD-II (78%), 81 HC (90%)	43.1 (11.3), 43.8 (12.4), 45.8 (12.1)	CTQ	GM volume	In BD patients, higher levels of childhood traumas were associated with lower volumes in the hippocampus
Song *et al*. (2020)	36 BD (55.6%), 29 HC (65.6%)	30.6 (8.3), 29.3 (5.3)	CTQ	GM volume	Compared to HCs, BD patients were characterised by negative correlations between CTQ scores and GM volumes in the right precentral gyrus and left middle frontal gyrus
Begemann *et al*. (2021)	248 BD (52%), 79 SCZ (35%), 216 HC (56%)	48.1 (12.2), 32.6 (11.1), 43.1 (14.5)	CTQ short form	GM volume	Patients with a history of childhood trauma shower lower GM volumes in the right medial orbitofrontal, paracentral, superior frontal regions and the left precentral area
Hsieh *et al*. (2021)	38 BD (68%)	37.2 (12.5)	CTQ	BOLD signal	Resting-state activity revealed that physical neglect was negatively correlated with left-caudate functional connectivity to the frontoparietal network, right supramarginal gyrus, left inferior parietal lobule, right middle frontal gyrus and right superior parietal lobule

BD, bipolar disorder; BD-I, bipolar disorder type 1; BDNF, brain-derived neurotrophic factor; BOLD, blood oxygenation level-dependent; CC, corpus callosum; CTQ, Childhood Trauma Questionnaire; GM, grey matter; HC, healthy controls; OFC, orbitofrontal cortex; PFC, prefrontal cortex; RFQ, Risky Families Questionnaire; SCZ, schizophrenia; WM, white matter.

Of all the included studies, 85% (12 out of 14) explored the neuro-structural correlates of CAs in BD (i.e. GM volumes, WM structural integrity) through magnetic resonance imaging and diffusion tensor imaging. Only a handful of studies (two out 14) explored the functional correlates of CAs in BD. Finally, two studies explored the association between brain-derived neurotrophic factor levels and CAs in BD patients showing that patients with a history of CA are marked by lower levels (Aas *et al*., 2014, 2019). The Childhood Trauma Questionnaire (CTQ) was used to assess and quantify CAs in the sample in 13 out of the 14 studies (92%) (Bernstein *et al*., 1994). Briefly, the CTQ is a retrospective self-report questionnaire assessing and quantifying CAs including neglect, physical, emotional and sexual abuses. It is composed of 70 items and responses are quantified on a five-point Likert scale according to the frequency with which CAs occurred (1 = ‘never true’ and 5 = ‘very often true’).

Studies investigating the association between CAs and neuro-structural alterations showed mixed findings. Higher levels of CAs correlated with the reductions of the hippocampal volumes in BD patients (Aas *et al*., 2013, 2014; Janiri *et al*., 2017, 2019) with only one study showing larger hippocampal volumes (Janiri *et al*., 2017). Specifically, BD patients with CAs were compared with other patients without a history of CAs (Janiri *et al*., 2017) or with HC (Janiri *et al*., 2019) or divided into two groups according to specific genetic vulnerabilities (Aas *et al*., 2013, 2014). Other structural studies investigating the possible GM correlates of CAs in BD patients showed volume reductions in the thalamus (Duarte *et al*., 2016; Poletti *et al*., 2016), alterations in the amygdala (Souza-Queiroz *et al*., 2016; Janiri *et al*., 2017) and in several frontal regions including the pre-frontal, orbito-frontal, para-central cortices and the middle-frontal gyrus (Duarte *et al*., 2016; Poletti *et al*., 2016; Song *et al*., 2020; Begemann *et al*., 2021). As shown in Table 1, these alterations seem to indicate that BD patients with a history of CAs are characterised by volume reductions in all these regions when compared with HC or similar group without a history of CAs. Lastly, studies investigating WM integrity showed an association between a history of CAs in BD and a reduction of volumes and fractional anisotropy (FA) in the CC, uncinate fasciculus and corona radiata when compared with HC or BD patients without CAs (Bücker *et al*., 2014; Souza-Queiroz *et al*., 2016; Stevelink *et al*., 2018).

Functional studies exploring the association between CAs and neuro-functional alterations in BD are scarce and show mixed findings employing both at-rest and task-related designs. For example, Souza-Queiroz *et al.* showed that, when compared with HC, BD patients with a history of CA are marked by lower pre-fronto-limbic connectivity at rest (Souza-Queiroz *et al*., 2016). This finding was partially replicated by another study by Hsieh *et al*. (2021) showing the presence of lower connectivity between left-caudate and the fronto-parietal network, right supra-marginal gyrus, left inferior parietal lobule, right middle frontal gyrus and right superior parietal lobule in BD patients with a history of physical neglect. Lastly, only a single study employed a task-related design showing that BD patients with a history of childhood trauma present stronger activations in the right angular, supra-marginal, middle temporal gyri and lateral occipital cortex during an emotion recognition task (Aas *et al*., 2017).

Overall, our review suggests that in BD patients, CAs are associated with GM alterations of the hippocampus, thalamus and across prefrontal regions (i.e. dorsolateral prefrontal, orbitofrontal cortices and precentral, middle frontal gyri). These alterations are expressed as volume reductions with only a paucity of studies showing opposite trends (Janiri *et al*., 2017). Moreover, patients with a history of CAs are also distinguished by alterations of the CC mainly expressed as volume or FA reductions. These results are not entirely surprising; in fact, the hippocampus has been previously shown to be altered in psychotic patients with a history of CAs, especially in the early phases of the condition (Hoy *et al*., 2012). Similar findings have been previously associated with cognitive dysfunctions in both BD and schizophrenia in several key cognitive domains including psychomotor speed, memory and executive functioning (Knöchel *et al*., 2014, 2016, 2017). Alterations of the hippocampus – the most consistent finding of our review – could be due to the fact that the hippocampus is part of the hypothalamus–pituitary–adrenal axis known to be influenced by stressful events and possibly leading to volume alterations (Heim *et al*., 2008). Notably, these alterations are known also for other psychiatric disorders such as post-traumatic stress disorder, schizophrenia and depression (de Kloet *et al*., 2006; Wingenfeld and Wolf, 2011; Belvederi Murri *et al*., 2014). Moreover, microglial activations – an indicator of neuronal damage – have been found in both thalamus and hippocampus in rodents exposed to stressors suggesting a specific effect of stress on mammals' brains (Sugama *et al*., 2007). Likewise, alterations of the thalamus, prefrontal regions and CC have also been suggested to be present in schizophrenic patients with a history of CAs, possibly indicating a transdiagnostic effect, linking both affective and non-affective conditions (Cancel *et al*., 2019). Notably, the thalamus is connected through sensory fibres with the amygdala playing an important role in filtering sensory information and regulating emotional responses (Herrero *et al*., 2002).

Several limitations should be considered when interpreting the results of our review. First, studies investigating the association between CAs and neurobiological variations from the norm are cross-sectional and retrospective. Moreover, the magnitude of the observed effects is unclear as none of the studies reported the effect size or the confidence intervals of the results. Lastly, only a handful of studies differentiated between different types of CAs or compared patients' group with and without a history of CAs. Therefore, future studies are warranted taking into account these limitations informing future diagnostic systems and interventions about the exact neurobiological firms of CAs in the BD brain.

## Data Availability

The data that support the findings of this study (search query) are available on request from the corresponding author.
